# *KRAS* and *NRF2* drive metabolic reprogramming in pancreatic cancer cells: the influence of oxidative and nitrosative stress

**DOI:** 10.3389/fcell.2025.1547582

**Published:** 2025-06-11

**Authors:** Valentina Rapozzi, Clara Comuzzi, Eros Di Giorgio, Luigi E. Xodo

**Affiliations:** ^1^ Department of Medicine, Laboratory of Biochemistry, University of Udine, Udine, Italy; ^2^ Department of Agricultural Food Environmental and Animal Science, University of Udine, Udine, Italy

**Keywords:** KRAS, *NRF2*, *NOS2*, metabolic reprogramming, PDAC, ROS, RNS

## Abstract

Cancer cells are subject to metabolic reprogramming, which leads to a sustained production of reactive oxygen species (ROS). Increased oxidative stress contributes to genomic instability and promotes malignant transformation. To counteract excessive ROS levels, cells activate nuclear factor erythroid 2–related factor 2 (*NRF2*), a key regulator of redox homeostasis that coordinates the transcription of a wide range of antioxidant and cytoprotective genes. This review examines the metabolic adaptations controlled by the *KRAS–NRF2* axis under oxidative stress conditions. In addition, we highlight a novel function of NRF2 in regulating the expression of *NOS2* by binding to a DNA enhancer element, thereby modulating the production of reactive nitrogen species (RNS). Finally, we discuss novel molecular strategies aimed at disrupting adaptive antioxidant responses in cancer cells and provide insights into combinatorial therapeutic approaches targeting redox balance in cancer.

## 1 Introduction

Pancreatic cancer is an extremely aggressive and deadly type of cancer that originates in the pancreas: an organ that lies behind the stomach and plays a crucial role in digestion and blood sugar regulation (Park et al., 2021). Among the different types of pancreatic cancer, pancreatic ductal adenocarcinoma (PDAC) is the most common, originating from the exocrine cells responsible for the production of digestive enzymes (Back et al., 2022). PDAC accounts for about 90% of all pancreatic cancers (exocrine and neuroendocrine cancers) and develops from the epithelial cells lining the ducts that transport digestive enzymes to the small intestine. PDAC is characterized by rapid progression and a high propensity to metastasize to nearby organs such as the liver and lungs (Ho et al., 2021) and is often only diagnosed at an advanced stage as it is asymptomatic in early stages. This late detection combined with resistance to conventional therapies contributes to the poor prognosis, with a 5-year survival rate of only ∼10%. It is predicted that this disease will be the second most common cause of cancer-related death in Western countries by 2030 (He et al., 2024). Somatic mutations play a key role in the development of PDAC, and *KRAS* is the most commonly mutated gene and found in over 90% of cases (Lennerz and Stenzinger, 2015). Mutations in *KRAS* lead to uncontrolled cell growth and contribute significantly to the progression of pancreatic cancer. Other frequently mutated genes are *TP53* (∼50–75% of cases), *CDKN2A* (p16), *SMAD4* (in ∼50% of cases), *BRCA2* and *ATM* (Hahn et al., 1996; Lai et al., 2021; Schutte et al., 1997; Matrone et al., 2004).

A characteristic feature of PDAC cells is their increased oxidative stress compared to normal cells (Hayes et al., 2020). This results from an imbalance between the production of reactive oxygen species (ROS) and nitrogen oxide species (RNS) and the detoxification capacity of the cells. In cancer cells, mitochondrial dysfunction in the electron transport chain (ETC.) is the main cause that leads to an increase in ROS production (Hardie et al., 2017; Sarwar et al., 2022). Oncogenes such as *KRAS* and *MYC* also drive metabolic changes that further increase ROS levels. In addition, hypoxic regions in tumours are exposed to metabolic changes that also increase ROS production. ROS levels are mainly regulated by *NRF2*, the main transcription factor that controls the cell’s antioxidant defence system (Ma, 2013). Dysregulation of *NRF2* in cancer cells can create a delicate balance where ROS levels remain high enough to support tumour growth and survival but still within the cell’s tolerance threshold (Jung et al., 2018; Osman et al., 2023; Wu et al., 2020) This review examines the intricate interplay between ROS, oncogenic *KRAS* and *NRF2* in cancer, with a focus on metabolic reprogramming that occurs under oxidative stress conditions. This may provide valuable insights for the rational design of new therapeutic strategies for this disease, which tends to be refractory to conventional treatments.

## 2 Sources of oxidative and nitrosative stress

### 2.1 Sources of ROS in cancer cells

**FIGURE 1:**
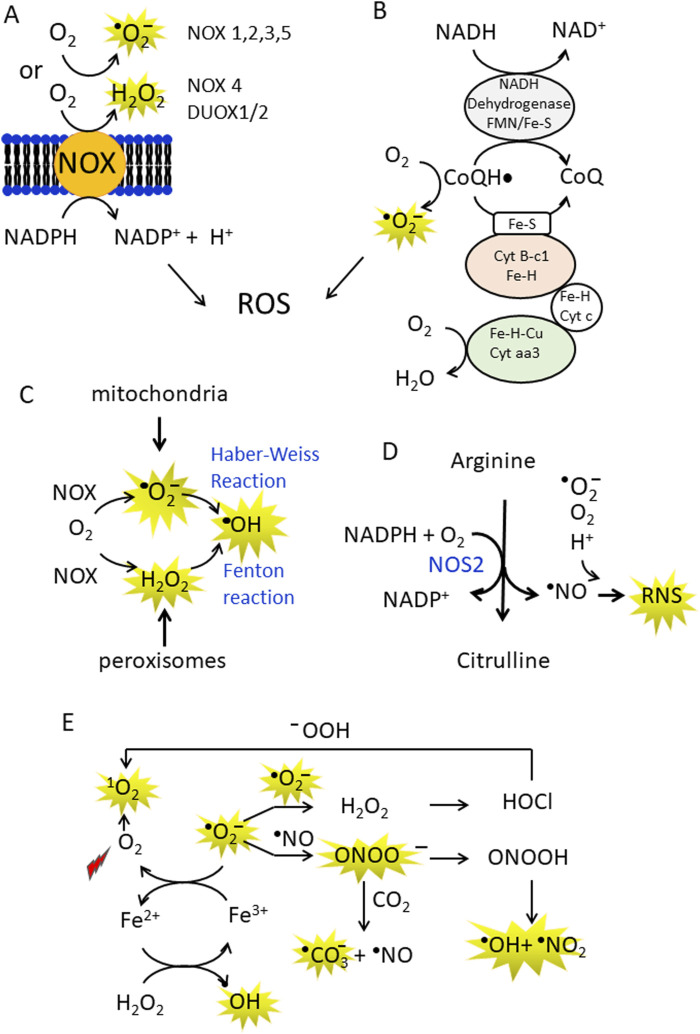
**(A,B)** Production of superoxide anions (•O_2_¯) and hydrogen peroxide (H_2_O_2_) by NADPH oxidases (NOX enzymes) and CoQ in the mitochondrial electron transport chain (ETC.). In the ETC some electrons are inadvertently transferred from CoQH^•^ to O2 in the mitochondria to generate •O_2_¯; **(C)** •O_2_¯ can be non-enzymatically converted to the more reactive hydroxyl radical (•OH) in the Haber-Weiss reaction. In addition, metal ions such as Fe^2+^, Cu^+^ can act as single electron donors in the Fenton reaction to give •OH; **(D)** Arginine is the substrate of NOS2, which converts arginine to citrulline, releasing ^•^NO and RNS; **(E)** A conversion between ROS and RNS occurs in the cell. At high concentrations, ^•^NO can combine non-enzymatically with •O2¯ and form peroxynitrite (ONOO¯). Peroxynitrite is a strong oxidising agent that is stable and can diffuse through membranes and interact with proteins (methionine and -SH groups). It can also split into the hydroxyl radical and nitrogen dioxide (NO_2_). In addition, ONOO¯ can interact with CO_2_ to form the carbonate anion and ^•^NO. The superoxide anion can be converted into H_2_O_2_and O_2_ by spontaneous or enzymatically controlled dismutation. Hydrogen peroxide via Fenton reaction is transformed in •OH or to hypochlorous acid (HClO) by myeloperoxidase. HClO produces singlet oxygen, a strong oxidising agent, in the presence of hydroperoxides.

The mitochondrial, ETC., and nicotinamide dinucleotide phosphate (NADPH) oxidases (NOX) are the main sources of ROS in the cell (Figures 1A,B) (Skonieczna et al., 2017). In addition, cytochrome P450 and xanthine oxidase, that transfer electrons from NADPH to O_2_ via FAD and heme cofactors, produce superoxide (^•^O_2_^−^) and hydrogen peroxide (H_2_O_2_) (Parvez et al., 2018). In the ETC, coenzyme Q (CoQ) plays a central role in the generation of the superoxide anion (^•^O_2_^−^). This occurs because during the transport of electrons from NADH/FADH_2_ to oxygen, some of them escape when the semiquinone CoQ^•^ accidently transfers its electron to O_2_ and forms ^•^O_2_^−^, a reactive but relatively short-lived oxygen radical (Sohal and Forster, 2007). ROS are also generated by mitochondrial dysfunction induced by mutated *KRAS*. For example, *KRAS G12V* disrupts mitochondrial function, reducing oxygen consumption and increasing ROS production (Hu et al., 2012). Downregulation of NDUFAF1, a factor for mitochondrial complex I assembly, has been linked to reduced mitochondrial respiration in *KRAS*-related cancers (Wang et al., 2015). Mitochondrial production of ^•^O_2_^−^ is estimated to be <0.2% of the total O_2_ consumed by the organelle (St-Pierre et al., 2002). Superoxide is rapidly detoxified by mitochondrial superoxide dismutase (SOD) to hydrogen peroxide (2 ^•^O_2_^−^+2H^+^ → H_2_O_2_ + O_2_) or can cross the mitochondrial membrane via VDAC, a voltage-dependent anion channel (Tikunov et al., 2010; Madesh and Hajnóczky G., 2001). Hydrogen peroxide easily enters the cytosol via the membrane aquaporin (Tafani et al., 2016), but can also be converted into H_2_O and O_2_ by catalase (2 H_2_O_2_ → 2 H_2_O+ O_2_). The superoxide anion can also react through non-enzymatic Haber-Weiss and Fenton reactions to form hydroxyl radicals, the most reactive ROS (Kehrer JP, 2000; Thomas et al., 2009) (Figure 1C). It has been demonstrated that hypoxic conditions exacerbate oxidative stress mainly by disrupting mitochondrial function and increasing ROS production (D’Aiuto et al., 2022). The exact mechanisms by which hypoxia increases ROS levels are still unclear, but there is evidence that hypoxia can increase ROS production by impairing complexes I, II and III of the, ETC (Kondoh et al., 2013; Wang et al., 2007). In addition, cancer cells often exhibit impaired antioxidant defence mechanisms, e.g., reduced glutathione levels or decreased activity of SOD and catalase, making them more susceptible to oxidative damage (Niu et al., 2021). The neutrophils and macrophages present in the tumour microenvironment are also a source of ROS (Wu et al., 2020; Wang et al., 2021).

### 2.2 Cancer cells produce nitric oxide

Cancer cells produce nitric oxide (^•^NO) and reactive nitrogen species RNS (Xu et al., 2002). The main source of ^•^NO in the cells are the nitric oxide synthases (Föstermann and Sessa, 2012) (Figure 1D). Of the three known isoforms, inducible NOS (iNOS or NOS2) can increase the aggressiveness of pancreatic cancer cells (Wang et al., 2016). NOS2 contributes to higher ^•^NO production under inflammatory and hypoxic conditions (Franco et al., 2004). Elevated ^•^NO levels are often associated with tumour progression, metastasis and chemoresistance. These levels are generally observed when iNOS is highly expressed due to inflammatory signalling (Fukumura et al., 2006). An important aspect of ROS/RNS is that they interconvert each other (Figure 1E). The superoxide anion produced by the NOX enzymes and the ETC can rapidly dismutate spontaneously or enzymatically to H_2_O_2_ and O_2_. The spontaneous dismutation has a rate constant of 10^5^ M^-1^s^-1^ (Sheng et al., 2014), but the enzymatic reaction is four orders of magnitude higher and therefore extremely efficient, with a kinetic constant comparable to the diffusion rate. However, superoxide can react with ^•^NO at a high rate (10^10^ M^-1^s^-1^) to generate peroxynitrite ONOO¯, a powerful oxidising agent (Fridovich, 1983). Although this RNS species intrinsically decays to ^•^OH and ^•^NO_2_ (Radi, 2013a; Radi, 2013b), it can modify proteins, oxidise thiols and react with fatty acids to generate reactive electrophilic species (Bartesaghi and Radi, 2018). The peroxynitrite species can undergo a one-electron reaction with CO_2_ to produce the carbonate ^•^CO_3_^−^ and ^•^NO_2_ radicals (Denicola et al., 1996). Despite the carbonate radical is less oxidising than ^•^OH, it reacts with amino acids with a high second-order rate constant (10^6^–10^8^ M^-1^s^-1^) (Bonini and Augusto, 2001). Finally, H_2_O_2_ is converted to HOCl in neutrophils by myeloperoxidase. HOCl can react with the peroxide ion to form singlet oxygen ^1^O_2_ (Onyango AN, 2016). In cancer cells, both ROS and RNS are important activators of cell signalling and in the next paragraph we will take a closer look at cell signalling mediated by ROS.

## 3 ROS-mediated cellular signalling in cancer cells

### 3.1 Enhanced levels of H_2_O_2_ stimulate cell proliferation

**FIGURE 2:**
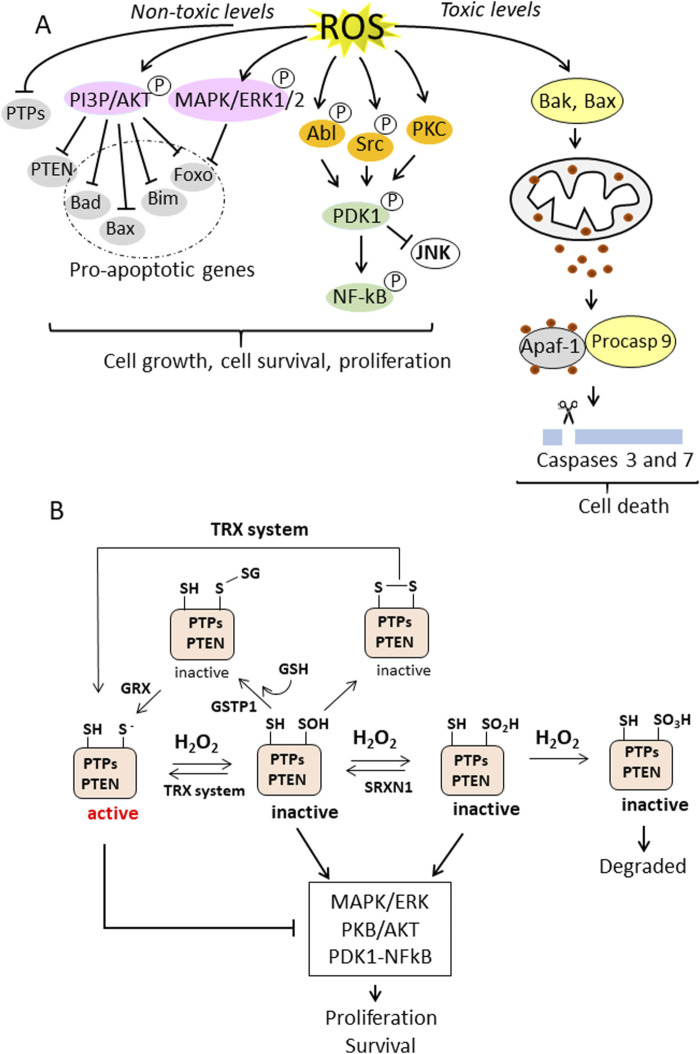
**(A)** ROS-induced cellular signalling in cancer cells. Non-toxic levels of ROS induce the phosphorylation and activation of PI3P/AKT and MAPK/ERK1/2 and the simultaneous inactivation of protein tyrosine phosphatases (PTPs) and lipid phosphatase, resulting in the inhibition of proapoptotic genes and the stimulation of cell growth and survival. ROS also activates the PDK1/NF-kB signalling pathway, leading to survival and proliferation. In contrast, overproduction of ROS leads to toxic oxidative stress, which activates Bak (Bcl-2 homologue antagonist/killer) and Bax (Bcl-2-associated X protein), which are pro-apoptotic members of the Bcl-2 protein family that regulate apoptosis and in particular the intrinsic (mitochrondrial) apoptosis pathway. Bax and Bak form pores in the outer mitochrondrial membrane that allow the release of cytochrome c into the cytoplasm. Cytochrome c induces the formation of the apoptosome, which activates caspase 9, which in turn activates executioner caspases 3 and 7; **(B)** PTPs and PTEN have a Cys residue in the active site in a thiolate state, which is susceptible to oxidation. The thiolate can be oxidised to sulfenate (-SOH), sulfinate (-SO_2_H) or sulfonate (-SO_3_H) depending on the H_2_O_2_ concentration. The Cys in the active site can also form disulfides, either with GSH, a reaction catalysed by GSTP1, or with another thiol in the active site, forming a disulphide bridge. These oxidative modifications inactivate the phosphatases and thereby enhance the MAPK/ERK and PKB/AKT pathways. The oxidative inactivation to sulfenate or sulfinate can be reversed by the antioxidant systems TXN1 or SRXN1. This restores phosphatase activity and promotes suppression of the MAPK/ERK and PKB/AKT signalling pathways. Similarly, Cys in the active site that have formed S-glutathionylation or have formed a disulfide bridge can be reversed by the enzyme glutaredoxin (GRX) (catalyses the reversible reduction of glutathione-protein mixed disulfide) or the TRX system. The oxidation of Cys in the active centre to a sulfonate state is irreversible (right) and the altered protein is degraded.

Due to its relatively longer half-life and higher diffusivity compared to superoxide and the hydroxyl radical (10^−3^ s for H_2_O_2_ compared to 10^−9^ s for ^•^OH and 10^−6^ s for ^•^O_2_^−^) (Rubio and Ceron, 2021), H_2_O_2_ is the most important ROS in cell signalling. Since H_2_O_2_ is generated from various sources, including ^•^O_2_^−^dismutation, NOX and xanthine oxidase enzymes and fatty acid oxidation in peroxisomes, it is the most abundant ROS in cells, including pancreatic cancer cells (Liu et al., 2023). There is clear evidence that the increase of ROS in cancer cells stimulates cell growth and survival (Hayes et al., 2020; Storz, 2016). This occurs by inhibiting the activity of protein tyrosine phosphatases (PTPs), the phosphatase and tensin homologue (PTEN) and MAPK phosphatases, thereby activating the MAPK/ERK, PI3P/PKB/AkT and PKD1/NFkB signalling pathways (Moloney and Cotter, 2018; Song et al., 2005) (Figure 2A). These PTPs phosphatases (PTP1B, PTPN2, PTPN11 and PTEN) are characterised by an active site containing a thiolate group susceptible to oxidation. Elevated H_2_O_2_ concentrations, as in cancer cells, oxidise the cysteine thiolate to sulfenate (SOH), sulfinate (SO_2_H) or sulfonate (SO_3_H), depending on the H_2_O_2_ content and duration of the ROS exposure (Figure 2B). The oxidative modification of the cysteine residue in the catalytic site of the phosphatases leads to their inactivation and thus to an increase in MAPK/ERK, PI3P/PKB/AKT and PKD/NFkB signaling, which promotes cell growth and survival. In the oxidised state at sulfenate, the catalytic site can be further inactivated by forming a disulfide bridge with either reduced glutathione (GSH) or with another cysteine in the catalytic site, leading to an increase in growth pathways (Hayes et al., 2020). These modifications are reversible as the disulfide can be reversed by the antioxidant systems thioredoxin (TXN1)/thioredoxin reductase (TXNRD1) or sufiredoxin 1 (SRXN1) (Hayes et al., 2020). This leads to activation of phosphatases and blocking of the MAPK/ERK, PI3P/PKB/AKT and PKD/NFkB signalling (Figure 2B). However, it should be noted that increased ROS levels are beneficial to cells if the risk of ROS-induced death is controlled by antioxidant systems. In other words, the ROS content should not be too high, otherwise the cells will die by apoptosis or other types of death instead of proliferating. Therefore, tumour cells upregulate antioxidant transcription factors and reprogram the metabolism to increase the levels of NADPH and GSH. That ROS in non-toxic concentrations are beneficial for cancer growth was shown in the study by Song et al. (2018), which demonstrated that suppression of ROS by N-acetylcysteine (NAC) reduced lung carcinoma in a *KRAS G12D*-driven mouse model.

### 3.2 High levels of ROS activates apoptosis

Overproduction of ROS is toxic to the cell and induces cell death by apoptosis (Zhang et al., 2016). Superoxide ^•^O_2_^−^produced in the, ETC., can impair mitochondrial functions (Kondoh et al., 2013) and cause mitochondrial membrane damage with leakage of cytochrome c from the intermembrane space. In the cytoplasm, cytochrome c induces the activation of caspase 9 and executioner caspases 3 and 7 (Figure 2A) (Walsh et al., 2008). These caspases cleave PARP-1 into two fragments of 24 and 89 kDa, which are no longer able to perform DNA repair functions. These conditions favour DNA fragmentation, chromatin condensation and membrane blebbing, the hallmarks of apoptotic cells. (Mills et al., 1998).

High levels of ROS can also activate the extrinsic apoptotic pathway, known as the death receptor pathway, which is triggered by the binding of signals from outside the cell such as tumour necrosis factor-alpha (TNF-α) or Fas ligand (FasL) to their respective death receptors (Fulda and Debatin, 2006). After TNF-α and FasL bind to the death receptors, procaspase-8 (or procaspase-10, depending on the cell type) is recruited to the death receptor. The recruited procaspase-8 is autocatalytically cleaved and converted to active caspase-8, which initiates the downstream cascade by direct cleavage and activation of executioner caspases (Kantari and Walczak, 2011).

In the next section, we will discuss how the KRAS-NRF2 axis controls ROS homeostasis and induces a metabolic shift in cancer cells.

## 4 The interplay between ROS, *KRAS*, and *NRF2* in redox homeostasis and metabolic reprogramming in pancreatic cancer

### 4.1 NRF2 controls redox homeostasis

Cancer cells preferentially convert glucose into lactate even when sufficient oxygen is available: a phenomenon known as the Warburg effect (Vander Heiden et al., 2009). This metabolic shift is in stark contrast to normal cells, which under aerobic conditions mainly use oxidative phosphorylation (OXPHOS) to maximise ATP production. Since aerobic glycolysis yields only 2 ATP per glucose molecule compared to the 36 ATP generated by full glucose oxidation via glycolysis, tricarboxylic acid cycle and OXPHOS, it is upregulated in cancer cells to provide biomass for rapid proliferation and adapt to hypoxic conditions (Gatenby and Gillies, 2004; Zhou et al., 2022).

There is increasing evidence that metabolism in pancreatic cancer cells is controlled by the oncogenic *KRAS*, which activates several downstream signalling pathways. In particular, the PI3K/AKT signalling pathway increases glucose uptake (Hong et al., 2016; Fontana et al., 2024) and glycolysis (Hu et al., 2016), while the MAPK/ERK signalling pathway stimulates cell proliferation (Drosten and Barbacid, 2020) and modulates the expression of metabolic enzymes (Papa et al., 2019). In early tumour stages, the blood supply is reduced, leading to hypoxic conditions. Hypoxia inducible factor 1 (H1F-1a) is upregulated by the *KRAS* signalling pathway, allowing cancer cells to adapt to hypoxia. HIF-1α increases anaerobic glycolysis by stimulating the expression of glycolytic genes (Zhu et al., 2024). High glycolytic flux also drives the pentose phosphate pathway (PPP), which is critical for the production of ribose-5-phosphate (a precursor for nucleotide biosynthesis) and NADPH. NADPH maintains redox balance and serves as coenzyme for NOX enzymes, particularly NOX4, which is overexpressed in pancreatic cancer cells and is a major source of oxidative stress (^•^O_2_^−^) (Ju et al., 2017). The control of redox homeostasis is critical for cancer cell survival and is primarily regulated by the nuclear factor erythroid 2-related factor 2 (NRF2) (Ngo and Duennwald, 2022). NRF2 activates the expression of various antioxidant genes − including superoxide dismutase (*SOD)*, catalase, glutathione peroxidase (*GPx*) and haemoxygenase-1 (*H O -1*) − and prevents excessive accumulation of ROS in the cell. Low or moderate ROS levels act as signalling molecules that regulate cell growth, differentiation and survival, while excessive ROS damage DNA, proteins and lipids and impair cell viability (Srinivas et al., 2019) (Figure 2A). NRF2, a member of the NFE2 family of transcription factors, plays a crucial role in redox homeostasis. Under non-stressed conditions, NRF2 is sequestered in the cytoplasm by its inhibitor KEAP1. KEAP1 promotes the ubiquitination of NRF2 and subsequent proteasomal degradation. When cellular ROS increase, as in the case of cancer cells, KEAP1, which contains several cysteine residues that are essential for its interaction with NRF2, is oxidised. The oxidation of the cysteines alters the conformation of KEAP1 and reduces its affinity for NRF2 (Wakahayashi et al., 2004). This allows NRF2 to escape proteasomal degradation and migrate to the nucleus, where it acts as a transcription factor by binding to the antioxidant response element (ARE), thereby activating the expression of antioxidant genes. When the oxidative stress decreases, KEAP1 is restored to its reduced state and resumes its function as a target for NRF2 degradation. This dynamic regulation ensures that NRF2 activation is tightly controlled and only occurs during cellular stress. Mutations in KEAP1 have been identified in patients with PDAC (Lister et al., 2011). Loss of KEAP1 function leads to abnormal activation of NRF2, which promotes the progression of PDAC. Conversely, depletion of NRF2 has been shown to inhibit tumour progression in mouse models of PDAC and non-small cell lung cancer (Romero et al., 2017).

### 4.2 NRF2 causes a metabolic reprogramming in PDAC

**FIGURE 3:**
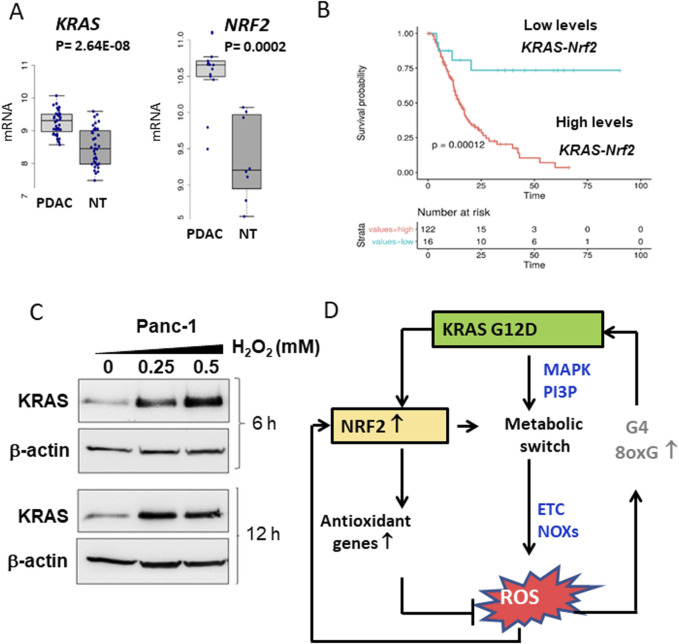
**(A)** Differential expression of *KRAS* and *NRF2* between normal and tumor tissues in PDAC patients. Data obtained from GSE15471; **(B)** Kaplan-Meir plots show that patients with high levels of *KRAS* and *NRF2* expression exhibit a lower survival probability than patients with low *KRAS* and *NRF2* expression; **(C)** Levels of KRAS and actin in Panc-1 cells treated with increasing amounts of H_2_O_2_; **(D)** Scheme showing the relationships between KRAS, NRF2 and ROS in pancreatic cancer cells. Panels A,B,C adapted with permission (iScience 2023, 26, 108566).

NRF2 plays a central role also in metabolic reprogramming by activating glycolysis, PPP, glutathione synthesis, long-chain fatty acid and glutamine metabolism (Di Giorgio et al., 2023; Mitsuishi et al., 2012). Similarly, *KRAS* increases the expression of enzymes involved in glucose and glutamine metabolism in PDAC (Ying et al., 2012; Gaglio et al., 2011). This functional synergy between *NRF2* and *KRAS* supports the hypothesis that these oncogenes jointly reprogramme the metabolism of cancer cells. Both genes are upregulated in pancreatic cancer cells (Figure 3A). Analysis of 175 tumour tissues from The Cancer Genome Atlas (TCGA) revealed a positive correlation between mRNA levels of *KRAS* and *NRF2*. Furthermore, PDAC patients with high expression of *KRAS* and *NRF2* had significantly poorer survival than patients with lower expression (Di Giorgio et al., 2023) (Figure 3B). These results emphasise a functional link between *KRAS* and *NRF2*. Direct evidence for the regulation of *NRF2* by *KRAS* comes from studies showing that oncogenic *KRAS* stimulates *NRF2* expression (Ferino et al., 2020a; Tao et al., 2019; Yang et al., 2021). Overexpression *of KRAS G12D* or *KRAS G12V* in Panc-1 cells leads to increased *NRF2* levels, while silencing *KRAS* with specific siRNA leads to reduced NRF2 expression (Di Giorgio et al., 2023). Another important observation is that both *KRAS* and *NRF2* expression levels are modulated by H_2_O_2_, further linking their regulation to oxidative stress (Figure 3C). All these data are consistent with the hypothesis that the regulation of metabolism and oxidative stress management in pancreatic cancer cells is related to the coordinated activity of *KRAS* and *NRF2*. This concept is illustrated in the diagram in Figure 3D. Mutant *KRAS G12D*, which occurs in ∼90% of PDAC, causes a metabolic switch in favour of aerobic glycolysis (Warburg effect), the pentose phosphate pathway (PPP) and increased glutamine metabolism (Ying et al., 2012). This reprogramming activates NOX enzymes, disrupts mitochondrial function and increases the production of reactive oxygen species (ROS), which promotes cancer growth. However, an excess of ROS can be cytotoxic, so *KRAS* upregulates *NRF2* via the PI3K/AKT and MAPK/ERK pathways. Under oxidative stress, NRF2 activates the expression of antioxidant genes and maintains ROS at levels that promote tumour proliferation. NRF2 also contributes to *KRAS*-driven metabolic reprogramming by promoting anabolic metabolism (Di Giorgio et al., 2023; Mitsuishi et al., 2012). For a comprehensive description of the metabolic reprogramming induced by the KRAS-NRF2 axis in conjunction with hypoxia we refer to reference Di Giorgio et al., 2023.

### 4.3 Mechanism by which ROS upregulate KRAS

Interestingly, ROS themselves stimulate the expression of both *KRAS* and *NRF2*. In the case of *KRAS*, the mechanism of ROS-mediated upregulation involves a G-rich promoter sequence upstream of the transcription start site (TSS). This region contains two G-quadruplex (G4) motifs that form folded secondary structures that recruit transcription factors (MAZ, PARP1 and hnRNPA1) (Cogoi et al., 2008). These G4 motifs are highly susceptible to oxidative modifications, in particular by the oxidation of guanine to 8-oxoguanine (Cogoi et al., 2018). Oxidation of the G-quadruplex structure facilitates the recruitment of transcription factors and increases *KRAS* transcription under oxidative stress. Taken together, these mechanisms reveal an intricate feedback loop in which *KRAS*, *NRF2* and ROS jointly regulate oxidative stress and drive metabolic reprogramming in PDAC cells. This interplay between *KRAS*, *NRF2* and ROS is shown schematically in Figure 3D. *KRAS G12D* stimulates PDAC growth by promoting a metabolic switch that drives anabolic processes and ROS production. To counteract the potentially cytotoxic ROS levels, *KRAS* activates *NRF2*, which not only attenuates oxidative stress but also promotes the metabolic shifts required for tumour growth. This dynamic interplay highlights the coordinated activity of *KRAS* and NRF2 in PDAC pathophysiology and emphasises their potential as therapeutic targets.

### 4.4 The suppression of NRF2 switches PDAC cells to aerobic metabolism

**FIGURE 4:**
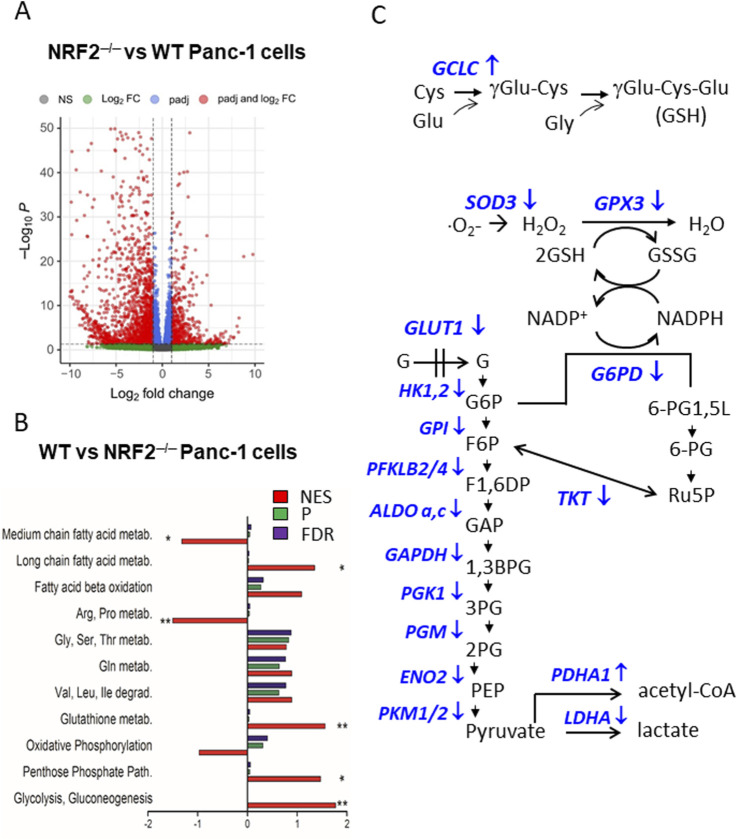
**(A)** Volcano plot of DEGs in Panc-1 NRF2^−/−^ cells compared to WT cells; **(B)** Functional enrichment analysis of WT Panc-1 cells compared to NRF2^−/−^ Panc-1 cells. This analysis shows that in cells where NRF2 was deleted (NRF2^−/−^), glycolysis, PPP, glutathione metabolism and long-chain fatty acid metabolism are inhibited, while arginine/proline and medium fatty acid metabolism are activated. NES = normal enrichment score; P = p-value; FDR = false discovery rate; **(C)** Glycolytic, PPP and glutathione metabolism enzymes are downregulated in NRF2^−/−^ cells (indicated with ↓). Panels A and B adapted with permission (iScience 2023, 26, 108566).

Recent research from our laboratory has further investigated the influence of *KRAS* and *NRF2* on the modulation of PDAC metabolism (Di Giorgio et al., 2023; 2024). *NRF2* was stably knocked down with CRISPR/Cas9 and two PDAC cell lines, Panc-1 and MIA-PaCa-2, without NRF2 (labelled NRF2^−/−^) were obtained. Using these edited cell lines, the cellular response was investigated under conditions in which the coordinated action of *KRAS* and *NRF2* is disrupted (Di Giorgio et al., 2023). RNA-seq analysis on Panc-1 NRF2^−/−^ cells (GEO: GSE217965) revealed 2,554 differentially expressed genes (DEGs) in NRF2^−/−^ cells compared to wild-type cells, with 1,888 downregulated DEGs and 666 upregulated DEGs, based on a threshold of |log_2_ FC| ≥ 1 and P < 0.05 (Di Giorgio et al., 2023) (Figure 4A). The functional enrichment analysis of DEGs with ClusterProfiler showed a significant decrease in glycolysis, the pentose phosphate pathway (PPP), the glutathione cycle and long-chain fatty acid metabolism as well as a simultaneous deep reactivation of arginine/proline and medium-chain fatty acid metabolism in NRF2^−/−^ Panc-1 cells compared to WT cells (Di Giorgio et al., 2023) (Figure 4B). These results suggest that NRF2 plays an important role in the switch of pancreatic cancer cells to aerobic glycolysis for ATP and biomass production as well as in the activation of PPP and glutathione cycle to maintain redox balance (Figure 4C). This is consistent with gene microarray data from two GEO datasets showing upregulation of glycolysis, PPP and GSH signalling pathways in NRF2-active oesophageal cells (Fu et al., 2019). Furthermore, silencing of NRF2 by siRNA in A549 lung cancer cells similarly reduced PPP enzymes and glutathione synthesis (Mitsuishi et al., 2012; Lu, 2009). Taken together, these data support the notion that NRF2 co-operates with *KRAS G12D* to shift glucose metabolism in PDAC towards aerobic glycolysis. Furthermore, recent work from our laboratory showed that the *KRAS-NRF2* axis regulates the expression of *NOS2* and the production of NO and RNS in PDAC: an argument that will be discussed in the next section (Di Giorgio et al., 2024).

## 5 *NRF2* regulates *NOS2* expression in pancreatic cancer cells

### 5.1 Suppression of NRF2 upregulates NOS2 in PDAC

**FIGURE 5:**
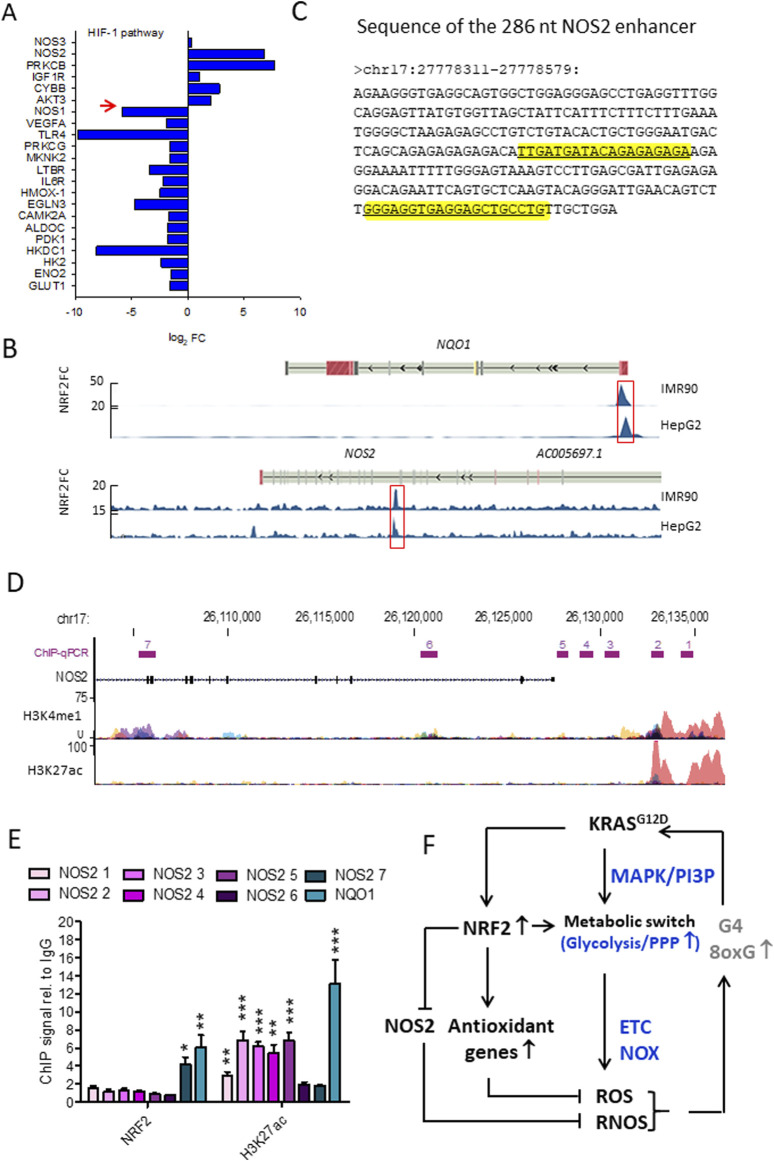
**(A)** Gene expression of the HIF pathway in NRF2^−/−^ cells. Note that *NOS2* is highly expressed in NRF2^−/−^ cells while *NOS1* is downregulated. The opposite holds for WT Panc-1 cells; **(B)** NRF2 ChIP-seq signals expressed as fold-change (FC) respect to Input in correspondence of *NQO1* and *NOS2* genomic loci. Data were retrieved from Encode and the significantly enriched peaks are highlighted; **(C)** Sequence of the NOS2 enhancer, the two predicted NRF2 binding sites are highlighted in yellow; **(D)** H3K4me1 and H3K27ac ChIP-seq signals expressed as fold-change (FC) compared to Input in correspondence of NOS2. The amplified regions investigated in qPCR are indicated (from 1 to 7), H3K4me1 and H3K27ac levels were obtained from Encode (cell lines: GM12878, H1-hESC, HSMM, HUVEC, K562, NHEK, NHLF); **(E)** ChIP signals relative to IgG obtained with anti-NRF2 and anti-H3K27ac antibodies in WT Panc-1 cells relative to the indicated genomic loci; **(F)** Interplay between KRAS, NRF2 and NOS2 in the control of oxidative and nitrosative homeostasis in PDAC. Panels A,B,C,D,E adapted with permission (BBA MCR 2024, 1871, 119106).

The functional enrichment analysis of DEGs in *NRF2* ^*−/−*^ Panc-1 cells compared to the WT cells showed a reactivation of the arginine/proline metabolism (Figure 4B). As arginine is a nonessential amino acid that serves as a substrate for nitric oxide synthase (NOS) enzymes, DEG analysis suggests that *NRF2*, and consequently *KRAS*, should control the expression of *NOS2* and nitric oxide (NO) production in PDAC cells. Gene Ontology (GO) analysis of DEGs in Panc-1 NRF2^**−/−**^cells compared to WT cells revealed four enriched terms involving *NOS2*, one of which is “HIF-1 signalling pathway (group P = 4.36 × 10^−3^). Considering its role in metabolic adaptation to oxygen and oxidative stress, analysis of the HIF signalling pathway showed that 5 genes were upregulated and 15 were downregulated, with *NOS2* being strongly upregulated (log_2_ FC = 6.84, P = 5.53 × 10^−6^) (Di Giorgio et al., 2024) (Figure 5A). GENEMANIA analysis of upregulated DEGs in the KEGG.HIF pathway (*NOS2, PRKCB, IGF1R, CYBB,* and *AKT3*) suggests interactions with genes encoding subunits of the enzyme complex that converts GTP to cGMP, a key second messenger in the NO-mediated signalling pathway. The downregulated DEGs in the HIF-1 pathway encode enzymes involved in anaerobic processes—glycolysis, PPP, and the glutathione cycle—suggesting that *NRF2* suppression renders Panc-1 cells dependent on aerobic metabolism. An important observation is that glycolytic Panc-1 cells, which rely primarily on glucose for ATP production and biomass synthesis, have low levels of NOS2 and NO, indicating that their metabolism is substantially dependent on arginine. In contrast, NRF2^−/−^ Panc-1 cells have approximately 4-fold higher levels of NOS2. This increase is related to their dependence on aerobic metabolism and altered arginine utilisation. Measurements of ^•^NO levels using DAF-FM DA, a non-fluorescent molecule that reacts with ^•^NO to produce a fluorescent benzotriazole, confirm that NRF2^−/−^ Panc-1 cells indeed contain more ^•^NO than wild-type (WT) cells (Di Giorgio et al., 2024). When NRF2 expression is restored in NRF2^−/−^ Panc-1 cells, NO levels decrease to the level of WT cells. This rescue experiment demonstrates that the regulation of NOS2 and ^•^NO in pancreatic cancer cells is controlled by NRF2, or more comprehensively, the KRAS-NRF2 axis. Further evidence for NOS2 activity in NRF2^−/−^ Panc-1 cells is provided by the citrulline/arginine ratio, which is 6-fold higher in NRF2^−/−^ cells compared to WT cells (Di Giorgio et al., 2024). Restoring NRF2 expression lowers this ratio to WT levels. Similarly, treatment with the NOS2 inhibitor 1400W equalizes the citrulline/arginine ratio between WT and NRF2^−/−^ cells, highlighting that NOS2 activity is dependent on NRF2 (Di Giorgio et al., 2024).

### 5.2 NRF2 binds to NOS2 enhancer and inhibits transcription

To investigate the mechanism by which NRF2 regulates *NOS2*, NRF2 ChIP-seq data from IMR90 (ENCSR197WGI) and HepG2 (ENCSR488EES) cells, available through ENCODE (Encyclopedia of DNA Elements), revealed that NRF2 interacts with a distal enhancer located 22 kb downstream of the NOS2 transcription start site (Figure 5B). Enhancers are short DNA sequences that increases the transcription of target genes. Unlike promoters, which are positioned directly adjacent to the genes they regulate, enhancers can influence gene expression from a great distance (Panigrahi and O’Malley, 2021). They recruit transcription factors and cofactors that facilitate the assembly of the transcriptional machinery at the promoter of the target gene. The NOS2 enhancer is 269 nt long and harbors two putative antioxidant response elements (AREs) recognized by NRF2 (Figure 5C) (Di Giorgio et al., 2024). Evidence that NRF2 binds to *NOS2* in Panc-1 cells was obtained by ChIP experiments using antibodies anti-NRF2 and anti-H3K27ac in which 7 sites of *NOS2* in genomic regions where histone H3 is epigenetically modified were amplified (Di Giorgio et al., 2024). The results showed that NRF2 binds to a genomic region of *NOS2* (site 7, Figure 5D) where histone H3 is epigenetically modified by methylation and not acetylation (Figures 5D,E). It should be remembered that histone H3 acetylation at lysine 27 (H3K27ac) is a well-characterized histone modification linked to active gene transcription and is often used as a marker to distinguish active enhancers from poised or inactive ones (Creyghton et al., 2010). This modification is catalysed by histone acetyltransferases (HATs), which transfer an acetyl group to lysine, which loses its positive charge and weakens the interaction between histones and DNA. The relaxed chromatin structure makes the DNA more accessible to transcription factors and other components of the transcription machinery, which promotes gene activation. When H3K27ac marks enhancer regions, it signifies an active state, as opposed to enhancers marked only by H3 methylation H3K4me1, which can be other in an active or in a poised/inactive state (Creyghton et al., 2010). The presence of H3K27ac at enhancers often facilitates the recruitment of coactivators, transcription factors, and mediator complex components, which collectively enhance transcriptional activation of nearby genes by stabilizing RNA polymerase II at the promoter region (Kang et al., 2021; Yao et al., 2020). The DNA region within the *NOS2* locus to which NRF2 binds to is marked by a lack of H3K27 acetylation (H3K27ac-) and a presence of H3K4 monomethylation (H3K4me1+) in wild-type Panc-1 cells (Figure 5D, site 7). This epigenetic profile indicates a repressed chromatin state with little or no transcriptional activity. At the same time, the presence of H3K4me1+, which indicates monomethylation at lysine 4 of histone H3, is usually associated with potential enhancer activity rather than active transcription. Taken together, these markers—H3K27ac- and H3K4me1+— represent a poised, not fully active state of the NOS2 enhancer in WT Panc-1 cells. In summary, NRF2 inhibits the transcription of *NOS2*. The interplay between *KRAS G12D*, *NRF2* and *NOS2* in the control of oxidative and nitrosative homeostasis in pancreatic cancer cells is summarised in Figure 5F. Given that NO and RNS are important contributors to the pathogenesis and progression of PDAC, in the next section, we discuss the effects of NO and RNS on pancreatic cancer cells.

## 6 Effect of nitric oxide on PDAC cells

Enhanced levels of NO/RNS have been observed in PDAC (Wang and Xie, 2010; Wang et al., 2016). NO acts as a signalling molecule in various physiological and pathological processes (Lowenstein and Padalko, 2004). NOS2 expression is triggered by inflammatory cytokines (TNF-α and IFN-γ) and hypoxic conditions in the tumour microenvironment. NO plays a dual role in tumour biology, influencing tumour growth, angiogenesis, metastasis and immune responses.

### 6.1 Dual role of NO in cancer cells

At low concentrations, ^•^NO promotes cancer cell survival and proliferation by activating the PI3K/AKT and MAPK/ERK1/2 signalling pathways (Ding et al., 2021). It also (i) stimulates the expression of vascular endothelial growth factor (VEGF), promoting angiogenesis and tumour growth (Dulak et al., 2000); (ii) modulates cell adhesion molecules and metalloproteases, enhancing cancer cell migration and invasion (Carreau et al., 2011); (iii) facilitates immune evasion by inhibiting cytotoxic T-cells and natural killer (NK) cells (Sato et al., 2007; Cifone et al., 2001). At high concentrations, ^•^NO has cytotoxic effects through the formation of RNS such as peroxynitrite, which triggers DNA damage and apoptosis in cancer cells (Virag et al., 2003). At high concentrations, NO can induce cell cycle arrest and inhibit proliferation of pancreatic cancer cells. To prevent the accumulation of toxic levels of ROS and RNS, PDAC cells upregulate *NRF2*, the master regulator of cellular redox homeostasis (Lister et al., 2011). NRF2 activation lowers ROS levels and, as recently reported, inhibits *NOS2*, the primary source of ^•^NO and RNS in cells (Di Giorgio et al., 2024). The upregulation of *NRF2* helps cancer cells maintain redox balance, which supports their survival and continued proliferation even under oxidative stress conditions.

### 6.2 Canonical and non-cnonical NO signalling pathways and protein S-nitrosylation

**FIGURE 6:**
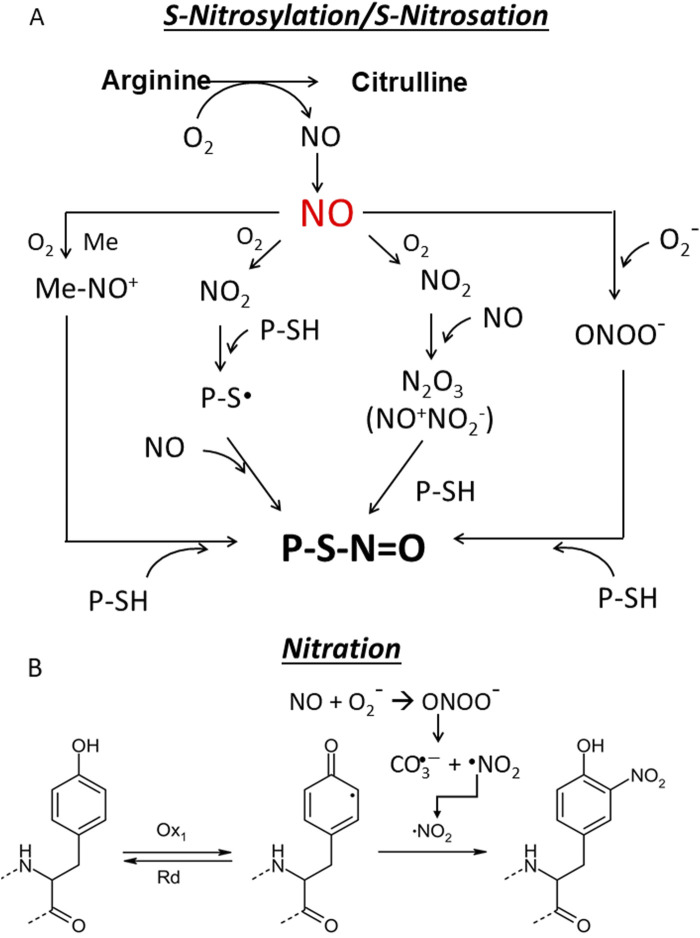
**(A)** S-nitrosylation/S-nitrosation. This cellular modification can occur in different ways. First, NO can form with O_2_ dinitrogen trioxide N_2_O_3_ which isomerises to nitrosonium nitrite (NO^+^NO_2_^−^) (Zakharov and Zakharov, 2009) whose nitrosonium NO^+^ reacts with a protein thiol (P-SH) to produce a nitrosothiol (P-S-N=O). In another pathway NO is oxidized to NO_2_ which reacts with a thiol group to give a thiol radical (-S∙) that with NO provides a nitrosothiol. The third pathway is mediated by metal which generates with NO a nitrosonium (NO^+^) (M^n+1^ + NO + O_2_→ M^n^-NO^+^), that adds to the thiol group to form a nitrosothiol (Aboalroub and Al Azzam, 2024; Ye et al., 2022; Reactive cysteines of proteins and glutathione (GSH) can undergo S-nitrosation by the peroxynitrite ONOO¯ to form S-nitrosothiol derivatives, P-SNOs and S-nitrosoglutathione (GSNO), respectively. Not all Cys are susceptible to S-nitrosylation. Those that are subject to S-nitrosylation lie within a consensus sequence that includes amino acids that create a hydrophobic environment. S-nitrosylation depends on several factors, including the acid/base and hydrophobic residues in the vicinity of the cysteine and the accessibility of the solvent (Marino and Gladyshev, 2010; Doulias et al., 2010). Notably, a hydrophobic environment attracts hydrophobic gases like NO and O_2_ and strongly enhances the rate of *S*-nitrosylation (Möller et al., 2007). S-nitrosation refers to a chemical process that occurs under physiological and pathological conditions in which peroxinitrite ONOO^−^ reacts non-enzymatically with the thiol group to form a nitrosothiol (P-S-N=O); **(B)** Nitration. The one-electron oxidation of tyrosine produces a tyrosine radical Tyr^•^, which is converted into 3-nitrotyrosine by reaction with ^•^NO_2_. As the pK value of the −OH group of Tyr is 10.3, it is 100% protonated at physiological pH, while the pK value drops to 7.3 during nitration and the -OH group is almost 50% deprotonated. This can strongly influence the structure of the nitrated protein.

Nitric oxide signalling pathways are divided into canonical and non-canonical pathways based on their mechanisms and targets. The canonical ^•^NO signalling involves the activation of soluble guanylyl cyclase (sGC) and the production of cyclic guanosine monophosphate (cGMP), which acts as a second messenger and activates downstream targets such as protein kinase G (PKG), phosphodiesterases (PDEs) and ion channels (Francis et al., 2010). At low NO levels (<100 nM), the cGMP/PKB pathway can protect against apoptosis and promote cell survival (Yasuhiro et al., 2006). At higher concentrations (>400 nM), NO activates non-canonical signalling by direct interaction with proteins leading to apoptosis. *S*-nitrosylation refers to a reversible modification in which ^•^NO binds covalently to a cysteine thiol and forms a nitrosothiol -S-N=O group (Figure 6A) (Hess et al., 2005; Jaffrey et al, 2001). Extensive S-nitrosylation of proteins has been associated with various diseases, including pancreatic cancer (Tan et al., 2019). This post-translational modification can affect function, stability, localisation and interaction of proteins and often contributes to carcinogenesis by altering important signalling pathways (Plenchette et al., 2015). Since excessive S-nitrosylation can be detrimental, cells have developed systems to denitrosylate proteins. Thioredoxin and glutathione, for example, remove ^•^NO groups from proteins, restoring free thiols and maintaining redox homeostasis (Sengupta and Holmgren, 2013). The biological role of S-nitrosylated proteins in PDAC, adjacent non-cancerous tissue, and Panc-1 cells was analysed separately (Tan et al., 2019). Functional analysis in non-cancerous tissues revealed that S-nitrosylated proteins are primaraly associated with basic biological processes, including primary cell metabolism, regulation of biological quality, response to stress and stimuli, catabolic activities, oxidation-reduction processes, secondary metabolism and initiation of translation. However, in PDAC tissues and Panc-1 cells, *S*-nitrosylated proteins were specifically enriched in pathways associated with tumourigenesis, such as cell cycle regulation, cell division, cell motility and actin filament-based processes. In addition, gene ontology annotations of cellular components revealed that *S*-nitrosylated proteins in these three samples are distributed in different subcellular compartments: cytoplasm, nuclear components, ribonucleoprotein complexes and ribosomes. These results emphasise the extensive involvement of *S*-nitrosylation of proteins in the pathogenesis of PDAC.

### 6.3 S-nitrosation and nitration of proteins

*S*-nitrosation is a chemical process occurring under physiological and pathological conditions where peroxinitrite ONOO^−^ reacts non-enzymatically with the thiol group, forming a nitrosothiol (-S-N=O) (Figure 6A). It is often used interchangeably with *S*-nitrosylation. This chemical reaction occurs on proteins and non-protein molecules such as glutathione GSH which is transformed into S-nitrosoglutathione (GSNO): a molecule acting as a NO reservoir in the cell (Ye et al., 2022). S-nitrosation of cysteines in the catalytic site of enzymes is a biologically important modification because it can alter metabolic pathays (Bruegger et al., 2018).

The term nitration refers to the addition of a nitro group (-NO_2_) to a tyrosine residue or other aromatic amino acids in proteins to form nitrotyrosine. Like S-nitrosation, it requires a peroxynitrite (ONOO-), which is formed by the reaction of NO with superoxide anion (^•^O_2_^−^). It is often associated with oxidative and nitrosative stress (Radi, 2013a) (Figure 6B). Nitration can irreversibly alter the structure and function of proteins and dysregulate signalling pathways (Bandookwala and Sengupta, 2020). Nitration is used as a biomarker for oxidative damage and inflammation in diseases such as atherosclerosis and neurodegenerative diseases. The characteristics of ^•^NO signalling pathways are summarised in Table1.

**TABLE 1:** Nitric oxide signaling.

Feature	S-Nitrosylation	Nitration	S-Nitrosation
Target	Protein CysteineThiol	Tyrosine residues	Thiol-containing molecules
Product Formed	S-nitrosothiols (R-SNO)	Nitrotyrosine (R-Tyr-NO_2_)	S-nitrosothiols (R-SNO)
Reversibility	Reversible	Irreversible	Reversible
Biological Role	Signaling	Marker of oxidative/nitrosative stress	Signalling

The level of *S*-nitrosylated proteins in cells is primarily regulated by enzymatic processes that ensure that *S*-nitrosylation occurs in a controlled manner to maintain homeostasis and signaling. The main source of *S*-nitrosylation is ^•^NO, but also S-nitrosoglutathione (GSNO), a low molecular weight S-nitrosothiol, can serve as ^•^NO donor for protein *S*-nitrosylation. GSNO acts as a stable ^•^NO reservoir and ensures the availability of NO for S-nitrosylation under physiological conditions (Broniowska et al., 2013). Key enzymes such as nitrosylases and thioredoxin/thioredoxin reductase (Trx/TrxR) and S-nitrosoglutathione reductase (GSNOR) precisely regulate the level of *S*-nitrosylated proteins in the cells (Anand and Stamler, 2012; Sengupta and Holmgren, 2012). Trx/TrxR reverses S-nitrosylation in proteins by catalysing denitrosylation, while GSNOR degrades GSNO and thus reduces the pool of available ^•^NO donors. This dynamic balance between S-nitrosylation and denitrosylation allows cells to fine-tune the levels of *S*-nitrosylated proteins in response to physiological and pathological conditions.

### 6.4 NO donors in cancer therapy

Given that ^•^NO at high concentrations arrests the cell cycle and inhibits proliferation, the use of ^•^NO donors has gained increasing interest in therapeutic applications. The therapeutic potential of ^•^NO was first explored many years ago, particularly in the treatment of pulmonary hypertension (Abman, 2013). However, due to the challenges associated with handling this gaseous molecule, ^•^NO donors present an attractive alternative. These compounds can generate ^•^NO in a controlled manner and have shown promise in cancer therapy. Wink and colleagues (Thomas et al., 2009) investigated the dose-dependent effects of ^•^NO on human breast cancer MCF7 cells. At low concentrations (1–30 nM), ^•^NO activates cyclic guanosine monophosphate (cGMP); at 30–100 nM, ^•^NO phosphorylates AKT; and at 100–300 nM, ^•^NO stabilizes hypoxia-inducible factor 1-alpha (HIF1α). At these relatively low concentrations, ^•^NO promotes proliferation and survival. However, at concentrations exceeding 1 μM, ^•^NO induces nitrosative stress, leading to cytostatic and apoptotic effects. Several NO donors have been proposed for therapeutic applications, offering a range of properties and effectiveness, including N-nitroso compounds as diazeniumdiolates, (NONOates); 3-morpholinosydnonimide (SIN-1) generating peroxynitrite and polymeric N-nitrosamines (Huerta, 2015; Mondal et al., 2024). For a comprehensive review of their characteristics, readers are referred to specific studies (Bhowmik and Roy, 2024; Huang et al., 2017; Huerta et al., 2008; Huerta S, 2015).

## 7 ChiP-seq analysis in 3D Panc-1 spheroids confirm *NOS2* regulation by NRF2

### 7.1 NRF2 controls NOS2 in Panc-1 sheroids

The regulation of *NOS2* expression by NRF2 was also investigated in Panc-1 spheroids, which are 3D cell models that better mimic the structural and biological features of tumours than 2D culture cells (Di Giorgio et al., 2024). H3K27ac-ChIP-seq analyses of WT and NRF2^−/−^ Panc-1 spheroids revealed significant acetylation in over 13,500 genes. Specifically, 569 genes showed acetylation in NRF2^−/−^ spheroids, while 734 genes showed acetylation in WT NRF2^+/+^ spheroids. Genes with increased acetylation in NRF2^−/−^ spheroids are primarily associated with signalling pathways involving arginine metabolism, epithelial-mesenchymal transition (EMT) and ^•^NO signalling (Di Giorgio et al., 2024). The regulatory role of NRF2 on NOS2 through the binding to a DNA enhancer was confirmed by mapping the H3K27ac signal at the *NOS2* locus, which showed a marked increase in acetylation at the distal enhancer region in NRF2^−/−^ spheroids compared to WT spheroids. The increased acetylation extended to a regulatory element located 5 kb upstream of the *NOS2* transcription start site (Di Giorgio et al., 2024). These findings reinforce the conclusion that NRF2 directly represses *NOS2* expression by modulating the chromatin state at its enhancer region in 3D spheroid model.

**FIGURE 7:**
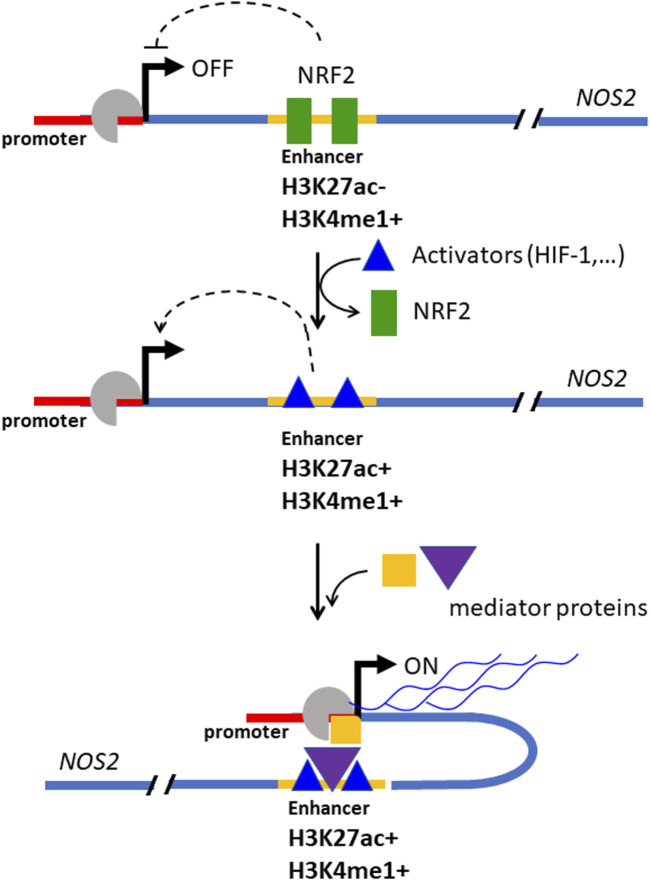
Mechanism by which NRF2 suppresses the expression of *NOS2* in Panc-1 cells. At elevated concentrations, NRF2 binds to a distal enhancer, inactivating it. The enhancer bound by NRF2 is locked to the activators protein and the expression of *NOS2* is inhibited (in this state the DNA at the *NOS2* locus is marked by H3K27ac- and H3K5me1+). When the NRF2 level decreases, the enhancer is unlocked, free of NRF2, and is bound by activator proteins that promote transcription (in this state the DNA at the *NOS2* locus is marked by H3K27ac+ and H3K5me1+). (“Med” stands for Mediator, a protein complex involved in gene expression in eukaryotic cells; “ac” = activator protein, TSS = transcription start site. Adapted with permission (BBA MCR 2024, 1871, 119106).

In addition to NRF2, hypoxia is a critical regulator of *NOS2* transcription. Suppression of NRF2 permits the release of the distal enhancer, facilitating the recruitment of transcriptional activators. The chromatin at the *NOS2* locus is characterized by the presence of H3K4me1—a mark of poised enhancers—indicating partial accessibility. Under hypoxic conditions, histone H3 becomes further acetylated at lysine 27 (H3K27ac), promoting chromatin relaxation and enhancer activation. Concurrently, levels of HIF-1 increase, leading to its binding at hypoxia response elements (HREs) within the *NOS2* locus, further enhancing transcription. As shown in Figure 7, a three-step mechanism for the full activation of *NOS2* has been proposed: (i) release of the enhancer from NRF2 inhibition; (ii) H3K27 acetylation leading to chromatin decompaction at the *NOS2* locus; and (iii) binding of activating protein to the enhancer and HIF-1 to the *NOS2* promoter (Matrone et al., 2004), forming the transcriptional complex.

### 7.2 Epigenetic mechanisms regulationg NOS2

Epigenetic mechanisms that regulate *NOS2* expression have been previously documented. For example, De Andrés et al. (2013) demonstrated that abnormal *NOS2* expression in osteoarthritic human chondrocytes is associated with DNA methylation changes in *NOS2* promoter and enhancer regions. Specifically, these authors reported demethylation of an NF-κB enhancer located −5.8 kb upstream of the transcription start site of *NOS2* gene. These authors demonstrated that demethylation of this enhancer is essential for *NOS2* transactivation. A comparable epigenetic regulatory mechanism was observed in human macrophages, where *NOS2* expression differs significantly from that in mouse macrophages (Ross et al., 2014). While mouse macrophages express high levels of *NOS2*, human macrophages produce low levels of *NOS2* and ^•^NO due to extensive DNA methylation near the transcription start site of human *NOS2*. In contrast, the mouse *NOS2* gene has low methylation in the 5′-flanking CpG regions. Analyses of chromatin accessibility and histone modifications showed that human macrophages have a closed chromatin conformation at the *NOS2* locus, whereas mouse macrophages have an open chromatin state that facilitates *NOS2* expression. This work emphasises how epigenetic changes control species-specific gene expression (Ross et al., 2014). Dreger et al. (Dreger et al., 2016) further investigated *NOS2* regulation by showing that cytokines strongly induce *NOS2* expression in rodent endothelial cells but not in human endothelial cells. *NOS2* was identified in human umbilical vein endothelial cells as a potential target of the histone methyltransferase EZH2, which catalyses the tri-methylation of histone H3 at lysine 27 (H3K27me3). The EZH2-mediated modification represents an epigenetic mechanism of gene silencing.

Another notable mechanism affecting chromatin accessibility at the *NOS2* promoter involves the NLRC4/caspase-1 axis in human macrophages (Buzzo et al., 2017). Caspase-1 cleaves PARP-1, a protein traditionally associated with apoptosis but also known to alter chromatin states essential for gene expression (Hottiger, 2015). Normally, PARP-1 maintains chromatin in a condensed state and suppresses gene expression. However, under stress conditions, PARP-1 is displaced from chromatin by caspase-1-mediated cleavage, leading to localised decondensation and allowing transcription factors to access DNA and promote gene expression (Erener et al., 2012).

Collectively, these studies highlight the multifaceted role of epigenetic mechanisms—including DNA methylation, histone modifications and chromatin remodelling—in regulating NOS2 expression in different cell types and species. In the following section, we report that NOS2 also plays a critical role for growth in NRF2^−/−^ Panc-1 spheroids.

## 8 PDAC-spheroids formation depends on NOS2: therapeutic implications

### 8.1 NOS2 strongly affect the growth of NRF2−/− spheroids

The role of NOS2 in the proliferation of Panc-1 cells was examined by comparing NRF2^−/−^ Panc-1 cells, which exhibit elevated NOS2 levels, to wild-type (WT) Panc-1 cells, which have lower NOS2 expression (Di Giorgio et al., 2024). To effectively suppress NOS2, esiNOS2—a pool of small interfering RNAs (siRNAs) targeting NOS2—was applied to both cell types. The impact of NOS2 knockdown on 3D spheroid formation was evaluated under conditions of both normal and suppressed NOS2 expression. Strikingly, NOS2 silencing had minimal impact on spheroid formation in WT Panc-1 cells, but resulted in more than a 50% reduction in spheroid formation in NRF2^−/−^ Panc-1 cells (Di Giorgio et al., 2024). These results underscore the metabolic dependence of NRF2^−/−^ Panc-1 cells on NOS2, and suggest that NOS2 is a potential therapeutic target. ^•^NO, at non-toxic levels (<100 nM), is a key regulator of cellular metabolism (López-Sánchez et al., 2020; Zhou et al., 2019). In Panc-1 cells, ^•^NO stabilizes HIF-1α, a transcription factor that upregulates glycolytic enzymes such as hexokinase (HK), phosphofructokinase (PFK), and pyruvate kinase (PK) under hypoxic conditions (Di Giorgio et al., 2024; Semenza, 2013). This promotes glycolysis as a compensatory mechanism for reduced mitochondrial respiration. Additionally, ^•^NO activates signalling pathways that support cell growth and survival (*vide infra*), suggesting that NOS2 inhibition—and consequently reduced ^•^NO production—may hinder PDAC progression. This notion is supported by recent findings from Reddy et al. (2024), who demonstrated that metaplastic breast cancer—a chemoresistant subtype with few treatment options—becomes sensitized to PI3K inhibitors when co-treated with NOS inhibitor L-NMMA. Building on the data presented here, a promising therapeutic strategy for PDAC may lie in targeting both NOS2 and the KRAS–NRF2 axis, as elaborated in the following section.

### 8.2 Combination therapeutic strategies for PDAC

The observation that the inhibition of *NOS2* (and the arrest of ^•^NO production) blocks the growth of NRF2^−/−^ Panc-1 spheroids can be used in combined therapeutic approaches. This is supported by several important considerations: (i) *KRAS* is essential for the growth of PDAC. Its inhibition suppresses cell proliferation and induces apoptosis and ferroptosis (Miglietta et al., 2017; Ferino et al., 2020b; Di Giorgio et al., 2022); (ii) *KRAS* regulates *NRF2*. *KRAS* overexpression upregulating *NRF2* and *KRAS* inhibition downregulates *NRF2*; (iii) Therapeutic suppression of *KRAS* results in the downregulation of *NRF2* and consequently upregulation of NOS2. This leads to a metabolic switch from anaerobic glycolysis to aerobic metabolism. PDAC cells resist to the *KRAS*-targeted therapy by redirecting energy production to aerobic pathways in which arginine plays a crucial role, supporting the synthesis of phosphocreatine (an ATP buffer), polyamines and the production of NO/RNS through NOS2 (Di Giorgio et al., 2023). This metabolic adaptation was also evident in analysis of RNA-seq data from organoids derived from PDAC patients treated with FOLFIRINOX, a combination of chemotherapeutic agents (folinic acid, fluorouracil, irinotecan hydrochloride, oxaliplatin) used to treat tumours with *KRAS* mutations, including PDAC. A comparative analysis showed that genes that were suppressed in FOLFIRINOX organoids were also significantly suppressed in NRF2^−/−^ Panc-1 cells. The most strongly repressed genes belong to the KEGG categories “glycolysis” and “genes upregulated by oncogenic *KRAS*” (Di Giorgio et al., 2023). This suggests that the suppression of the *KRAS*-*NRF2* axis and the simultaneous activation of arginine-based metabolic pathways is also observed in PDAC patients undergoing FOLFIRINOX therapy.

These observations suggest that combination therapies targeting both *KRAS* and *NOS2* may outperform *KRAS*-targeted monotherapies. While *KRAS* inhibition blocks tumour growth, resistance may develop as cells adapt metabolically (they undergo a metabolic shift from anaerobic to aerobic arginine-dependent metabolism). The resistance is accompanied by increased *NOS2* and ^•^NO production, which promote tumour growth and reduce the effect of anti-*KRAS* drugs. So, a dual therapeutic approach combining KRAS inhibitors with NOS2 inhibitors could enhance the anti-tumour response. Another interesting combination could include KRAS inhibitors and arginine antagonists such as homoarginine, which reduces ^•^NO production by NOS2. This strategy could further improve the efficacy of targeted *KRAS* therapies. Future research should focus on exploring the metabolic consequences of NOS2 inhibition in PDAC cells, particularly in combination with KRAS-targeted treatments.

## 9 Conclusion

Over the last 2 decades, considerable progress has been made in understanding the effects of ROS/RNS on cancer biology, but many aspects remain unresolved. Since KRAS mutations (mainly G12D and G12V) are present in over 90% of PDAC cases, this oncogene is a major trigger of the disease and a primary target for rational drug development. Recent advances have yielded promising drugs targeting specific KRAS mutations, including KRAS G12C inhibitors (such as AMG510 and MRTX849) (Canon et al., 2019; Mahadevan et al., 2023) and inhibitors for KRAS G12D and KRAS G12V. However, despite significant efforts, the efficacy of these KRAS-targeting strategies remains a challenge, which is why KRAS was considered an undruggable target for many years.

More effective therapies can be developed by understanding the metabolic response of tumour cells to treatments. Pancreatic cancer cells, for example, are often found in a hypoxic microenvironment characterised by a specific genetic signature: overexpression and constitutive activation of KRAS, overexpression of NRF2 and HIF-1, and restricted expression of NOS2 (Di Giorgio et al., 2024). PDAC cells with this genetic profile are highly dependent on glucose for ATP production and biomass for proliferation. They produce ROS, mainly through NOX enzymes and electron leakage from the ETC, but remain below the toxic threshold thanks to the robust antioxidant defence of NRF2, which prevents ROS overload of cancer cells. In addition to controlling the cellular redox system, an increased NRF2 level favours binding to the NOS2 enhancer. This leads to inhibition of NOS2 expression and relatively low levels of ^•^NO and RNS in PDAC. As mentioned above, this carefully regulated balance of ROS and NO/RNS supports both cell proliferation and survival. In addition, the hypoxic conditions that characterise PDAC stimulate the expression of HIF-1 and thus the synthesis of glycolytic and PPP enzymes, adapting the cells to anaerobic metabolism (Di Giorgio et al., 2023).

When KRAS is targeted, the treatment not only downregulates or inhibits KRAS, but also reduces NRF2 expression. This leads to a profound metabolic reprogramming in which the cancer cells switch from an anaerobic to an aerobic metabolism, with arginine playing a crucial role in this transition (Di Giorgio et al., 2023). In addition, reduced NRF2 levels weaken the antioxidant defence, leading to an increase in ROS and favouring the dissociation of NRF2 from the NOS2 enhancer and thus the transcription of NOS2. This leads to an increase in the concentration of ^•^NO. At high concentrations, ^•^NO has a negative effect on metabolism, but at lower concentrations ^•^NO stimulates metabolic activity. This suggests that NOS2 and its substrate arginine are critical components for cancer cell survival and adaptation during anti-KRAS therapies. Although FOLFIRINOX does not directly target KRAS, this combination chemotherapy protocol which is primarily used to treat PDAC, also activates arginine metabolism, highlighting the role of metabolic reprogramming in the development of therapy resistance in pancreatic cancer. These results indicate that KRAS and NOS2 are promising candidates for a synthetic lethality approach. Combination therapies with small molecules or inhibitors targeting both KRAS and NOS2 may prove more effective than KRAS-targeted monotherapies alone. Future research will focus on exploring such combination strategies to improve treatment outcomes in pancreatic cancer.

## Glossary

mRNA: Messanger RNA
ATP: Adenosin triphosphate
PDAC: Pancreatic ductal adenocarcinoma
NADPH: Nicotinamide adenine dinucleotide phosphate
NRF2: Nuclear factor erythroid 2-related factor 2
NOS1: Neuronal nitric oxide synthase (also called nNOS)
NOS2: Inducible nitric oxide synthase (also called iNOS)
NOS3: Endothelial nitric oxide synthase (also called eNOS)
^•^NO: Nitric oxide
**ONOO** ^−^: Peroxynitrite
ROS: Reactive oxygen species
RNS: Reactive oxygen nitrogen species
H3K27ac: histone 3 lysine 27 acetylation
H3K4me1: Histone3 lysine 4 mono-methylation
HIF-1α: Hypoxia-inducible factor 1
DEG: Differentially expressed gene
KRAS: Kirsten ras gene
OXPHOS: Oxidative phosphorylation
DAF-FM DA: 4-Amino-5-methylamino-2′,7′-difluorofluorescein diacetate
ENCODE: Encyclopedia of DNA Elements
FAD: Flavin adenine dinucleotide
FMN: Flavin mononucleotide
GMPc: Cyclic guanosine monophosphate
sGC: Soluble Guanylyl cyclase
GTP: Guanosine triphospohate
IL-1: Interleukin-1
IL-1R1: Interleukin-1 receptor type I
INF-γ: Type II interferon
JAKs: Janus family kinases
LPS: Lipopolysaccharides
NF-kB: nuclear factor kappa-light-chain-enhancer of activated B cells
