# Correction: KRAS and NRF2 drive metabolic reprogramming in pancreatic cancer cells: the influence of oxidative and nitrosative stress

**DOI:** 10.3389/fcell.2025.1661525

**Published:** 2025-08-08

**Authors:** Valentina Rapozzi, Clara Comuzzi, Eros Di Giorgio, Luigi E. Xodo

**Affiliations:** ^1^ Department of Medicine, Laboratory of Biochemistry, University of Udine, Udine, Italy; ^2^ Department of Agricultural Food Environmental and Animal Science, University of Udine, Udine, Italy

**Keywords:** KRAS, NRF2, NOS2, metabolic reprogramming, PDAC, ROS, RNS

In the published article, there was a typo in the **Article title**. Instead of “KRAS and NRF2 drive metabolic reprogramming in pancreatic cancer cells: the influence of oxidative and nitrosatice stress” it should be: “KRAS and NRF2 drive metabolic reprogramming in pancreatic cancer cells: the influence of oxidative and nitrosative stress”.

The original article has been updated.

